# The Role of Long Non-Coding RNAs in Cardiovascular Diseases

**DOI:** 10.3390/ijms241813805

**Published:** 2023-09-07

**Authors:** Linh T. T. Le, Chan X. T. Nhu

**Affiliations:** Biotechnology Department, Ho Chi Minh City Open University, Ho Chi Minh City 70000, Vietnam; chan.nxt@ou.edu.vn

**Keywords:** long non-coding RNA, atherosclerosis, myocardial infarction, myocardial hypertrophy, heart failure, clinical application

## Abstract

Long non-coding RNAs (lncRNAs) are non-coding RNA molecules longer than 200 nucleotides that regulate gene expression at the transcriptional, post-transcriptional, and translational levels. Abnormal expression of lncRNAs has been identified in many human diseases. Future improvements in diagnostic, prognostic, and therapeutic techniques will be facilitated by a deeper understanding of disease etiology. Cardiovascular diseases (CVDs) are the main cause of death globally. Cardiac development involves lncRNAs, and their abnormalities are linked to many CVDs. This review examines the relationship and function of lncRNA in a variety of CVDs, including atherosclerosis, myocardial infarction, myocardial hypertrophy, and heart failure. Therein, the potential utilization of lncRNAs in clinical diagnostic, prognostic, and therapeutic applications will also be discussed.

## 1. Introduction

Cardiovascular diseases (CVDs) are a term encompassing a broad spectrum of pathologies. Globally, CVDs are the most prevalent causes of morbidity and mortality. An estimated 18.5 million people die annually, with a third of those fatalities occurring among individuals under 70 years old [1]. Due to changes in lifestyle and an increase in the mean lifespan, its prevalence continues to rise. Over 4 million individuals die from CVDs in Europe alone each year, accounting for 44% of all deaths [2,3]. A total of 11.5% of American adults (27.6 million) have been diagnosed with CVDs. In the 1950s, CVD was an uncommon disease in China [4]. However, between 1990 and 2015, its mortality increased significantly, by 21.4% and 70%, respectively [5]. It is crucial to investigate CVD molecular pathophysiology for identifying novel biomarkers and therapeutic applications in the future [6].

Despite the fact that the majority of the human genome (90%) is transcribed into RNA molecules, only 2% of these transcripts code for proteins, with approximately 20,000 protein-coding genes. These untranslated RNAs are known as non-coding RNAs (ncRNAs). Based on their length, these ncRNAs are divided into short and long classifications. Short ncRNAs include PIWI-interacting RNA, small interfering RNA (siRNA), and microRNA (miRNA). Typically, these short ncRNAs are highly conserved. Long ncRNAs (lncRNAs) have a length greater than 200 nucleotides and are poorly conserved. Since all ncRNAs exceeding 200 nucleotides are arbitrarily classified as lncRNAs, this group of molecules is extremely heterogeneous. On the database of NONCODE (http://www.noncode.org (accessed on 1 July 2023)), the human, mouse, and bovine lncRNA genes are listed at 102.783, 87.553, and 27.793, respectively.

The LncRNA disease database v2.0 (www.rnanut.net/lncrnadisease (accessed on 1 July 2023)) lists over 205,959 lncRNA-disease correlations, including CDVs. Much study has been undertaken on the involvement of lncRNAs in CVDs. Screening 16,044 lncRNAs to develop lncRNA profiles for the heart and 29 other tissues identified approximately 2353 cardiac-specific lncRNAs (14.7%). Of these, about 273 lncRNAs (1.7%) are five times more expressed than other tissues, and roughly 4828 lncRNAs (30.1%) are at least five times more abundant than the average in all tissue types [7]. Although dysregulation of lncRNAs has been found in both human and rodent models of CVDs, transferring those detections from animal to human applications should be conducted prudently since lncRNAs are poorly conserved across species.

Long non-coding RNAs are dysregulated in the development and progression of various CVDs, such as atherosclerosis, myocardial infarction, hypertrophy, cardiac remodeling, myocardial damage, and heart failure. Whereby we describe the existing understanding of lncRNAs in CVDs. Moreover, prospective diagnostic and therapeutic applications of lncRNA will be discussed further.

## 2. Long Non-Coding RNA Functions

**Figure 1:**
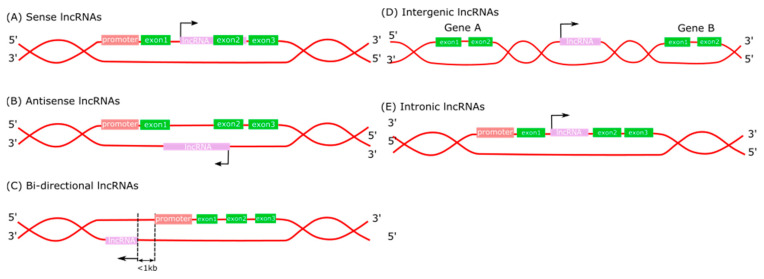
Classification of lncRNA.

Long non-coding RNA transcription start sites are marked by H3K4me3 histone modifications, while their transcriptional regions are marked with H3K36me3 [8]. LncRNAs are classified into five categories based on their relative position to protein-coding genes in the genome: sense lncRNAs, antisense lncRNAs, bidirectional lncRNAs, intergenic lncRNAs (lncRNAs), and intronic lncRNAs (Figure 1) [9,10]. Meanwhile, lncRNAs situated between two genes encoding proteins are divided into two main groups: enhancer-associated (elncRNA) and promoter-associated (lncRNA). ElncRNAs regulate the expression of adjacent genes encoding proteins on the same chromosome, whereas promoter-associated lncRNAs regulate chromosomal status and epigenetic inheritance [9,11]. Sense lncRNAs, transcribed from the sense strand, overlap with one or more exons of another protein-coding gene in the same strand [12,13]. Intronic lncRNAs typically begin and end in an intron of a protein-coding gene in either direction without exon overlap [14]. Antagonistic lncRNAs are transcribed from the complementary sequence of the gene encoding the protein. Thereby, they are located either on the same strand or the opposite strand of the nearest protein-coding genes and overlap with at least one protein-coding exon [14,15]. The outstanding point is that bidirectional lncRNAs are produced from the same promoter as gene-encoding proteins but in the opposite direction [16].

**Figure 2:**
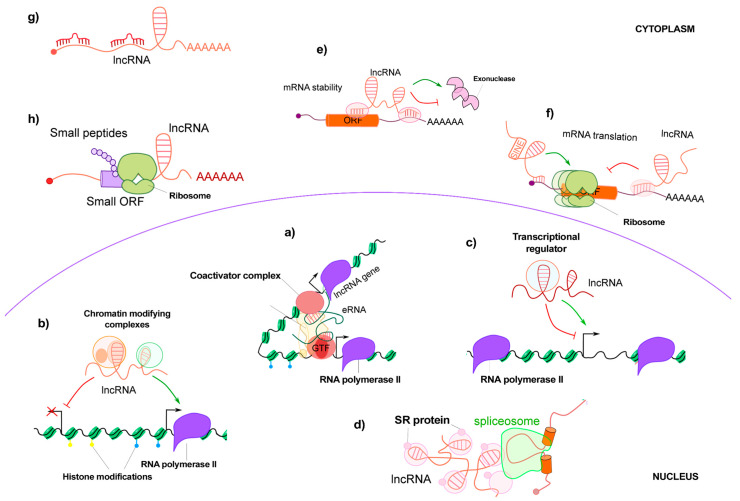
The function of long non-coding RNA. Nuclear lncRNAs can work as enhancer RNA (eRNA) (**a**), chromatin-modifying complexes (**b**), transcription factors (**c**), or affect pre-mRNA splicing (**d**). Cytoplasmic lncRNAs can regulate mRNA expression by affecting its stabilizing (**e**), translating (**f**), or competing for microRNA binding (**g**). Some can translate into active peptides (**h**).

Subcellular localization of lncRNAs plays a prominent role in their function. Long non-coding RNAs can affect transcription, post-transcription, and mRNA translation depending on their location. Nuclear-enriched lncRNA can change epigenetic genes to activate or suppress transcription. In contrast, cytoplasmic lncRNAs interact with miRNAs or proteins to affect protein localization, stability, and mRNA translation (Figure 2).

LncRNAs can enhance transcription (Figure 2a) or bind transcription factors to promoters to mediate histone modification (Figure 2b), which affect chromatin remodeling. For instance, by binding to components of the PRC2 (polycomb repressive complex 2) or WDR5/MML (WD repeat-containing protein 5/mixed lineage leukemia complex), lncRNA controls histone methylation: (1) PRC2-binding lncRNA FENDRR controls the level of histone methylation in the promoter regions of key transcription factors involved in myocardial development [17]; (2) Female mammals do not transcribe one X chromosome in early embryonic development. Xist will cause the X chromosome in its place to relocate to the outer edge of the nucleus. Afterwards, it interacts with PRC2 to elevate H3K27me3, resulting in X chromosome silencing and heterochromatinization [18,19] (3) HOTTIP recruits WDR5 to the promoter region of *HOXA*, the critical developmental gene, to activate its expression [20,21]. In addition, lncRNAs can block transcription factors from binding to the target region (Figure 2c). BHVT can bind to SUZ12, a PRC2 subunit, inhibiting PRC2 from binding to core genes of myocardial differentiation [22]. In addition to this, a number of lncRNAs function as transcription factors. For example, the lncRNA HSR1 can form a complex with the heat shock factor 1 (HSF1) and the eukaryotic translation initiation factor (eEF1A) to regulate the production of heat shock proteins in response to heat shock stress. In addition, they are able to control gene expression by having an effect on the splicing of pre-mRNA (Figure 2d). Through complementary pairing with mRNA, cytoplasmic lncRNA regulates mRNA post-transcriptional processes, thereby altering mRNA stability and translation (Figure 2e,f). For instance, TINCR and NR2F1-AS1 increase the stability and translational level of mRNAs through complementary binding to their target mRNAs [23,24]. By competitively binding to miRNAs, lncRNAs may operate as endogenous adsorbents to prevent miRNAs from destabilizing or interfering with the translation of their target transcripts, indirectly increasing mRNA transcription activity [25,26] (Figure 2g). For example, MEG3 regulates the expression of *ATF4* through endogenous competition for miR-214, thereby enhancing insulin resistance in the liver [27]. LncRNAs can also directly interact with proteins to regulate their functions. NORAD binds to PUMILIO to stabilize genomes. By binding to PUMILIO-related proteins needed for mitosis, DNA repair, and replication, NORAD maintains chromosomal segregation. In mice with coronary artery occlusion-induced myocardial infarction, lncRNA ZFAS1 is elevated in the cytoplasm and sarcoplasmic reticulum and directly binds to SERCA2a (Ca^2+^ATP enzyme 2a), inhibiting its activity. Furthermore, several lncRNAs include open reading frames (ORFs) that can be translated into biologically active short peptides (Figure 2h).

## 3. Functions of Long Non-Coding RNAs in the Cardiovascular System and Cardiac Development

Over 1000 tissue-, stage-, and heart-specific lncRNAs have been reported [28,29]. Among these, several lncRNAs are crucial to embryonic development and cardiac lineage commitment, e.g., FENDERR and BRAVEHEART [30,31]. FENDERR (fetal-lethal non-coding developmental regulatory RNA) has high expression in the germinal leaflet and lateral mesoderm, where it controls mesodermal differentiation, heart formation, and body wall formation. PRC2 and TrxG/MLL regulate chromatin state via FENDERR [32]. FENDERR−/− knockout mice had poor cardiac development and reduced embryo survival [33]. This highlights the importance of FENDERR for healthy cardiac growth and function. BVHT (Braveheart) plays an important role in the determination of the cardiac lineage in mice. It was found to be highly expressed in embryonic stem cells and essential for the maintenance of cardiac commitment [31,34]. BVHT governs the transition from nascent mesoderm to cardiac progenitor by regulating the core cardiovascular gene network and centrally mediating the epigenetic regulation of cardiac fate [31].

Additionally, a number of lncRNAs related to cardiac differentiation have been discovered. CARMEN (cardiac mesoderm enhancer-associated noncoding RNA) is also an integral regulator of human cardiac precursor cell fate and differentiation. CARMEN is similarly responsible for the cardiac specificity and differentiation of precursor cells by regulating the expression of PRC2. NKX2.5 is a vital transcription factor for cardiac differentiation and maturation. Expression of NKX2.5 is enhanced by the lncRNA Novlnc6, demonstrating its role as an enhancer in cardiac differentiation. Cardiomyocyte repolarization requires potassium fluxes controlled by Kv7.1 channels. These potassium channels, Kv7.1, are encoded by Kcnq1 [35]. During late embryonic development, Kcnq1 expression is regulated by the lncRNA Kcnq1ot1 (KQT-like subfamily, member 1 opposite strand/antisense transcript 1). This indicates that, at least during late embryonic development, this lncRNA is associated with cardiac contractile contraction.

## 4. Long Non-Coding RNAs and Cardiovascular Diseases

**Figure 3:**
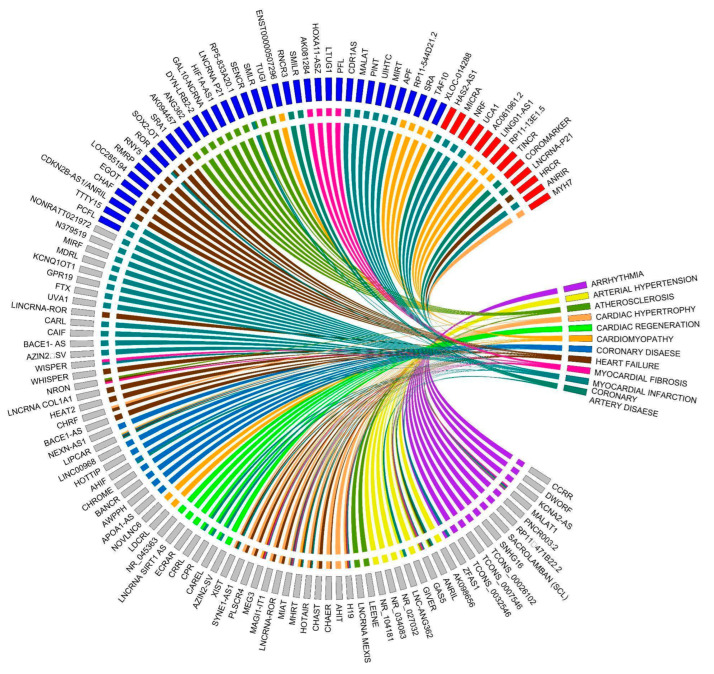
Long non-coding RNAs associated with cardiovascular diseases. The squares next to lncRNAs represent their expression levels. Red: increased expression; Blue: decreased expression; Gray: unidentified expression level.

**Figure 4:**
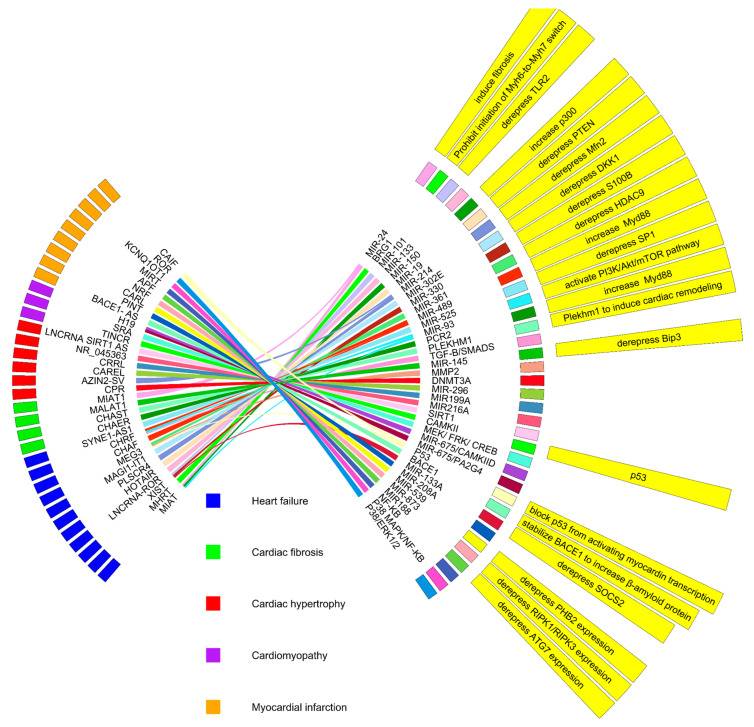
The role of some long non-coding RNAs in cardiovascular diseases. Long non-coding RNAs can either regulate the expression of target mRNAs or act as miRNA sponges to control the expression of target genes or signaling pathways, respectively.

Long non-coding RNAs are associated with various CVDs. In this review, we have summarized lncRNAs linked with CVDs such as arrhythmia, arterial hypertension, atherosclerosis, cardiac hypertrophy, cardiac regeneration, cardiomyopathy, coronary disease, heart failure, myocardial fibrosis, myocardial infarction, and coronary artery disease (Figure 3). To emphasize the significance of lncRNAs with specific functions in CVD, we will concentrate on lncRNAs associated with atherosclerosis, myocardial infarction, cardiac hypertrophy, and heart failure (Figure 4, Table 1). Overall, the mechanisms by which IncRNAs regulate pathophysiology remain elusive in the context of CVDs. However, these may be novel target molecules for the development of new diagnostic methods and therapeutic drugs for cardiovascular diseases.

### 4.1. Long Non-Coding RNAs in Atherosclerotic Vascular Diseases

#### 4.1.1. Atherosclerotic Vascular Diseases (ACDs)

Atherosclerotic cardiovascular disorders are the main cause of death worldwide.

In essence, atherosclerosis is a chronic progressive inflammatory process caused by a combination of pathological conditions, including dyslipidemia, cellular dysfunction of endothelial cells (ECs) and vascular smooth muscle cells (VSMCs), macrophages and other types of white blood cells, oxidative stress, and hemodynamic changes [79]. The disease is characterized by the formation and deposition of fibrofatty plaques in the walls of arteries, leading to the narrowing and stiffening of these vessels. Over time, blood flow is partially or completely blocked, resulting in tissue ischemia [80].

The pathogenesis of atherosclerosis begins with vascular endothelial damage. These lesions activate ECs, causing EC to proliferate. In addition, macrophages and other leukocytes, as well as platelets, infiltrate the affected arterial intima [81]. In addition, atherogenic cytokines and growth factors transform VSMCs from inactive to proliferative states. These aberrant changes result in the formation of fibrous tissue in the arteries. Abnormal lipid metabolism plays a significant role in the progression of the disease. Local deposition of lipids leads to the formation of fat streaks, thereby enhancing the formation of atherosclerotic plaque [82,83,84].

#### 4.1.2. Long Non-Coding RNAs and Atherosclerosis

Several LncRNAs are involved in the molecular mechanisms that regulate crucial signaling pathways in EC and VSMC cells, including apoptosis, autophagy, and proliferation. Furthermore, lncRNAs function as regulators of lipid homeostasis. Dysregulation of the expression of these lncRNAs is causally associated with the development and progression of CVDs, including atherosclerosis [85]. 

ANRIL, also known as CDKN2B-AS1, is approximately 3.8 kb in length and contains 19 exons from the Chr9p21 locus. Genome association studies identified that single nucleotide polymorphisms (SNPs) in this genomic region were directly associated with CVD risk and severity. Due to genetic variants in this region, 10% to 15% of the non-African population is susceptible to CVDs [86]. ANRIL expression is elevated in the atherosclerotic plaques of the patient. Several functions of ANRIL that have been determined include: (1) Modulating atherogenic cells by modulating CDKN2A/B [36]; (2) Acting as a sponge for many microRNAs, including miR-199a [37], miR-125a [38], miR-186 [39], and miR-323 [40]; (3) Being involved in the regulation of several signaling pathways, including ATM/E2F-1 [87], VEGF [88], and NF-κB signaling pathways [89]; (4) Relating to the immune response in EC cells; (5) Participating in the regulation of gene transcription by interacting with PRC2 [90]. On the promoter region and gene sequence of ANRIL, there are many Alu motifs. This sequence allows ANRIL to perform this function.

LncRNA TUG1 (Taurine-upregulated gene 1) is a Chr22q12-located, 6.7kb lncRNA. TUG1 functions as an endogenous molecular sponge for miR-133a, which depresses the expression of FGF1 (fibroblast growth factor). In addition to promoting cell proliferation and inhibiting apoptosis, TUG1 overexpression enhances the expression of inflammatory factors. Cotransfection with a miR-133 mimic eliminates these effects. This suggests that TUG1 exerts these effects at least via interaction with miR-133. On the other hand, TUG1 knockdown ameliorates atherosclerosis by reducing EC migration and adhesion to monocytes, stopping hyperlipidemia, reducing the inflammatory response, stopping neovascularization, and reducing the formation of atherosclerotic plaques. In ApoE−/− mice, administration of tanshinol to inhibit TUG1 results in a reduction in atherosclerotic lesions [41].

LncRNA H19 is shown to play two roles, both protecting and inducing atherosclerosis at different times during the formation and development of that disease. H19 is highly expressed in the serum of patients with atherosclerotic lesions. H19 promotes cell proliferation as well as reduces programmed cell death in VSMCs through two signaling pathways, MAPK and NF-κB pathways. Nevertheless, H19 also reduces vascular endothelial injury, thereby alleviating atherosclerosis. Another lncRNA, SMILR, also has the role of boosting the proliferation of VSMCs. SMILR is elevated in VSMCs stimulated with IL-1a and platelet-derived growth factor. Furthermore, SMILR levels are increased in atherosclerotic plaques. C-reactive protein and plasma SMILR are correlated.

LncRNA SRA is implicated in atherosclerosis formation. SRA enhances the expression of steroid receptor-dependent genes by interacting with multiple transcription factors [91], resulting in increased hypercholesterolemia and thereby enhancing the formation of atherosclerosis [91]. Atherosclerosis-promoting lncRNAs include DAPK-IT1. This molecule modulates the low-density lipoprotein (LDL) receptor by lowering statin responses to LDL and cholesterol and increasing their blood levels, promoting atherosclerosis [92]. Different from the previous lncRNAs, HOXC-AS1, which is also involved in lipid metabolism, inhibits oxLDL-induced cholesterol accumulation in the arterial intima by at least inhibiting the expression of HOXC6 [93]. Along with SRA, DAPK-IT1, and HOXC-AS1, lncRNA MEXIS is also involved in the lipid metabolism pathway. ABCA1 is a protein responsible for regulating cholesterol efflux and producing high-density lipoprotein (HDL). MEXIS augments ABCA1 expression and cholesterol efflux in macrophages [42]. Stable expression of MEXIS increases ABCA1 expression and significantly reduces the incidence of atherosclerosis in mouse models. On the other hand, MEXIS−/− mice decrease tissue-selective expression of ABCA1, and the probability of vascular occlusion is twice as high as that in normal mice.

Atherosclerosis is characterized by ECM remodeling and neointimal development. By activating the PI3K/AKT/mTOR pathway, lncRNA ENST00113 promotes neointimal formation and VSMC proliferation [94]. The deposition of hyaluronan also contributes to neoendothelial formation. Hyaluronan is synthesized by HAS2. Two independent lncRNAs that regulate expression of HAS2 are HAS2-AS1 (HAS2 antisense RNA 1) and SMILR. HAS2-A1 is elevated in atherectomy samples obtained from carotid arteries. HAS2-A1 promotes HAS2 transcription in VSMCs by modifying the chromatin structure in the presence of O-GlcNAcylation.

### 4.2. Long Non-Coding RNAs and Myocardial Infarction

#### 4.2.1. Myocardial Infarction (MI)

Myocardial infarction (MI) is the most deadly form of coronary atherosclerotic disease. An interruption in blood flow leads to ischemic processes as well as myocardial hypoxia. This phenomenon causes death and necrosis in the cardiomyocyte cells. If this condition persists in any coronary artery, a portion of cardiac muscle tissue will necrose [95]. Fibrotic remodeling in the infarct region is extremely significant for maintaining myocardial integrity and preventing cardiac wall rupture. The cardiac fibroblast (CF) cells need activation and differentiation to perform this cardiac remodeling. However, subsequent prolonged fibrosis increases cardiac stiffness, impaired cardiac function, and the development of heart failure [96]. In fact, myocardial injury following myocardial infarction (MI) almost always leads to adverse cardiac remodeling and fibrosis.

#### 4.2.2. Long Non-Coding RNAs and Myocardial Infarction (MI)

Multiple lncRNAs have abnormal expression in the pathophysiology of MI [85]. The aberrant expression of these lncRNAs may either aggravate or alleviate MI status. Although the function of these molecules is not thoroughly understood, their roles in cardiac cell homeostasis and the formation and development of disease have been identified. They may be involved in the regulation of autophagy, apoptosis, and oxidative stress in myocardial cells. However, it remains unclear whether altered expression of these lncRNAs is the cause or just the result of the disease. Much needs to be elucidated before these molecules can be exploited for clinical applications.

Similar to atherosclerosis, lncRNAs have also been identified as stipulating MI susceptibility. Specifically, in a genome-wide association study in the Japanese population, Ishii et al. identified the lncRNA MIAT (myocardial infarction-associated transcript) and its six genetic variants that conferred MI susceptibility [97]. Wherein, there was a variant associated with upregulation of MIAT expression. In addition to the disease-related genetic trait, MIAT was found to be overexpressed in both humans and mice with MI. This increased expression was directly related to infarct size in a mouse model of permanent occlusion of the left anterior descending artery [43]. Increased MIAT is detrimental to the heart because it plays a role in promoting cardiac fibrosis and adverse cardiac remodeling after MI. This conclusion was supported by several experimental results: (1) In an MI mouse model with increased MIAT, levels of TGF-β (transforming growth factor β), an important cytokine that promotes fibrosis, also increased dramatically; (2) MIAT acted as a sponge on miRNAs with anti-fibrotic function, such as miRNA-24, miR-29, miR-30, and miR-133, in the infarcted rat hearts [43]; (3) when performing knockdown MIAT by siRNA or lentiviral, cardiac function was improved, and post-MI cardiac fibrosis was also reduced. MIAT did this by controlling the proliferation and collagen production of CF cells.

Analogous to MIAT, WISPER, a lncRNA transcribed from the cardiac-specific super-enhancer region, is also highly expressed in the post-MI infarct region in mice and patients with aortic stenosis [98]. The level of WISPER correlates with the degree of cardiac fibrosis. Functionally, Wisper has been ascertained to be implicated in cardiac fibrosis by controlling the proliferation, migration, and apoptosis of CFs cells in vitro and in vivo. Furthermore, this molecule also controls the expression of pro-fibrotic factors such as COL3A1, FN1, and TGFB2. Resembling MIAT, an increase in WISPER is detrimental to the heart. Some empirical evidence helps confirm this, as follows: (1) When performing knockdown of WISPER with GapmeR, post-MI mice have smaller infarct sizes, reduce fibrosis, and preserve cardiac structure and function; (2) WISPER directly interacts with TIAR (TIA1-related) to regulate the alternative splicing of PLOD2 (procollagen-lysine, 2-oxoglutarate 5-dioxygenase 2), which catalyzes cross-linking lysyl hydroxylation reactions and collagen deposition [44] (3) Knockdown Wisper blocks transmembrane shuttling of TIAR protein to the nucleus and prevents TIAR-PLOD2 interaction.

Infarct areas will accumulate more activated CFs to release ECMs. H19 is overexpressed in CFs in the cardiac infarcted area in a mouse model of MI, suggesting that H19 is associated with fibrosis in MI. Gain- and loss-of-function studies have verified a role for this molecule in MI, in which H19 exacerbates post-traumatic fibrosis [45]. H19 acts by interacting with YB-1 (Y-box-binding protein 1), a protein involved in fibrosis, through inhibition of COL1A1 activity [46]. In MI, due to increased H19 expression, an H19-YB-1 complex is established. This leads to a rising expression of COL1A1, further promoting cardiac remodeling. In addition, H19 also interacts with PRC2 in cardiomyocytes. PRC2 mediates H3K27me3, an important transcriptional repressive marker of histones. The silence of H19 results in a global increase of H3K27me3 in conjunction with increased methylation enrichment of TESC (Tescalcin) and inhibition of its activity. TESC regulates the NFAT signaling pathway involved in angiogenesis. Therefore, the increase of H19 in MI will prevent the inhibition of the TESC locus, leading to inhibition of the NFAT (Nuclear factor of activated T-cells) signaling pathway.

Two lncRNAs were also found to be strongly elevated in the MI mouse model of coronary ligation, MIRT1 and MIRT2 (myocardial infarction-associated transcripts 1 and 2). Nevertheless, the expression of these two lncRNAs did not increase linearly with disease progression but was dynamically adjusted. In particular, their expression levels peaked immediately after the induction of myocardial infarction and returned to normal after 2 days. Currently, the expression level of these two lncRNAs in humans has not been determined because of their unidentified homologs. MIRT1 and MIRT2 play two roles, e.g., protective and harmful to the heart. They have a protective role in left ventricular function since their expression levels correlate with genes involved in the maintenance of the reserved ejection fraction and left ventricular remodeling after MI [47,48]. On the other hand, they may be harmful to the heart because MIRT1 harmonizes the inflammatory response after MI. Hypoxia-induced upregulation of MIRT1 in neonatal mouse CFs enhances the NF-kB signaling pathway and the proinflammatory cytokines, e.g., IL-6, IL-1β, and TNF-α. This results in enhanced apoptosis of cardiomyocytes and macrophage infiltration into the infarcted tissue. MIRT1−/− mice improved cardiac function because of a reduction in cardiomyocyte apoptosis and the infiltration of inflammatory cells into the heart.

Cellular homeostasis is maintained by the breakdown of cell proteins and organelles through autophagy. There is evidence suggesting that a certain degree of autophagy has a cardioprotective effect [99]. However, the accumulation of autophagosomes can lead to cardiomyocyte death, especially during post-ischemic reperfusion (I/R). Autophagy is found to be enhanced in some cardiovascular diseases [100]. Particularly for the process of cardiac remodeling, the disorder of autophagy is considered a disease trigger factor. As well, inhibiting the autophagic process may protect the heart from CVDs. Myocardin is a vital component of autophagy. Myocardin knockout will inhibit autophagy and reduce cardiac I/R injury. The cardioprotective role of the lncRNA CAIF (cardiac autophagy inhibitory factor) is manifested by its interaction with the p53 protein and its ability to prevent p53 from activating myocardin transcription [50]. Together with CAIF, the APF lncRNA also participates in the regulation of autophagy [49]. Increased APF due to injury induced by I/R conditions. This increase helps preserve myocardial function in response to the lesion caused by this process. APF enhances cardiac autophagy by binding to miR-188-3p as a ceRNA and leading to increased levels of ATG7, a miR-188-3p target gene and a factor promoting autophagy. Possibly, the increased APF helps to retain a certain degree of cell autophagy. From there, it may have a cardioprotective role. Overexpression of these lncRNAs in vivo results in smaller-sized infarcts. Another lncRNA molecule, XIST, has the ability to inhibit miR-133a and SOCS2 (suppressor of cytokine signaling 2) to facilitate the autophagic death of myocardial cells and exacerbate myocardial ischemic damage [51].

The loss of mature cardiomyocytes and their failure to regenerate is one of the causes that leads to cardiac dysfunction and post-traumatic heart failure [101]. Methods to regenerate cardiomyocytes and reactivate their proliferative potential will be an effective treatment for cardiovascular diseases [102]. Several lncRNAs associated with cardiomyocyte division may be viable targets for achieving this goal. Cardiac-specific lncRNA CAREL suppresses cardiomyocyte mitosis by competitively inhibiting endogenous miR-296, and thereby increasing the expression of miR-296 target genes TRP53INP1 and ITM2A, thereby inhibiting cell division. CAREL silencing promotes cardiac remodeling and improves cardiac function in neonatal and adult mice after MI [52]. Likewise, lncRNA CPR (cardiomyocyte proliferation regulator) is also involved in myocardial cell proliferation [53]. Knockout of CPR activates cardiomyocyte proliferation, promotes cardiac repair, and improves cardiac function after MI, while its overexpression has the opposite effect. The inhibitory effect of CPR on cardiac proliferation is related to its interaction with MCM3, which is the molecule that initiates DNA replication and the cell cycle. CPR interacts directly with the CpG site in the MCM3 promoter, promoting methylation that inhibits its expression and, consequently, cardiomyocyte proliferation. In addition, the two lncRNAs CRRL and AZIN2-sv inhibit cardiomyocyte regeneration by binding to miR-199a-3p and inhibiting the PI3/Akt signaling pathway, respectively [54,55].

In addition to their proliferative involvement, several lncRNAs have been shown to be relevant to the apoptosis of cardiomyocytes. The CARL (cardiac apoptosis-related lncRNA) liberates PHB2 from pre-apoptotic miR-539, leading to inhibition of mitochondrial fission and apoptosis of cardiomyocytes [56]. In fact, mice that increased CARL expression by adenovirus had a smaller infarct size. LncRNA MDRL (mitochondrial dynamic related lncRNA) decreased expression after MI [57]. Similar to CARL, MDRL was also able to inhibit mitochondrial fission and cardiomyocyte apoptosis through sequestering the pro-apoptotic miR-361. In the same way, increased expression of MDRL resulted in a smaller infarct size in the MI mouse model. As well as CARL and MDRL, FTX (Five Prime to Xist) was inhibited after I/R injuries. FTX can counter apoptosis through the regulation of the apoptosis repressor BCL2L2 via sequestration of miRNA-29b-1-5p in vitro [58]. UCA1 (urothelial carcinoma-associated 1) was a second LncRNA with expression altered in patients with MI [103]. In a rat model of I/R-induced heart injury, UCA1 contributed to cardiac damage by promoting cardiomyocyte apoptosis [104]. HOTAIR (HOX transcript antisense RNA) was another example of a lncRNA with cardioprotective functions in MI, having low expression in the plasma of mouse models and MI patients and in hypoxic cardiomyocytes. HOTAIR inhibits apoptosis by interacting with miRNA-1, releasing BCL2 from miR-1 inhibition, and thereby inhibiting the programmed cell death pathway [59]. Moreover, lncRNA ROR can aggravate MI by promoting the oxidative stress response and apoptosis pathway [105].

Besides apoptosis and autophagy, several lncRNAs are also involved in the necrosis of cardiomyocytes. For example, the necrosis-related factor (NRF) lncRNA can enhance cardiomyocyte necrosis. Knockdown of NRF antagonizes necrosis in cardiomyocytes and reduces necrosis and MI upon I/R injury. NRF functions by sequestering miR-873. This microRNA inhibited the translation of RIPK1/RIPK3, thereby inhibiting in vitro and in vivo H2O2-induced necrotic cell death as well as I/R injury-induced cardiomyocyte necrosis. During I/R injury, NRF was transcriptionally activated by p53. High expression of NRF increased binding to miR-873, thereby releasing RIPK1/RIPK3, resulting in H2O2-induced injury attenuation and reduced myocardial ischemic damage [60].

Several lncRNAs have been shown to be relevant to hypoxia-induced injury, or I/R injury. Overexpression of the lncRNA RMRP, a component of mitochondrial RNA processing endoribonuclease, aggravates hypoxia-induced injury in vitro. RMRP activates the PI3K/AKT/mTOR pathway in hypoxia-treated cells by capturing miR-206, which indirectly enhances the expression of the miR-206 target molecule ATG3. The lncRNA RMPR/miR-206/ATG3 axis plays a cardioprotection role against myocardial I/R injury [61,106]. Patients with MI had increased expression of the lncRNA aHIF (hypoxia-inducing factor) [62]. Under ischemic conditions, the lncRNA aHIF destabilized the HIF-1α mRNA, the master regulator of cellular responses such as angiogenesis and hypoxia [62,63]. Another lncRNA involved in myocardial I/R injury is MALAT1 (metastasis-associated lung adenocarcinoma transcript 1). MALAT1 was increased in reperfusion injury. Fentanyl has cardioprotective effects in myocardial I/R Injury by inhibiting MALAT1 and BNIP3 as well as increasing miR-145. In contrast, MALAT1 overexpression or miR-145 knockdown reversed the cardioprotective effects of fentanyl and exacerbated I/R injury [64].

### 4.3. Long Non-Coding RNAs and Cardiac Hypertrophy

#### 4.3.1. Cardiac Hypertrophy

Myocardial hypertrophy is an adaptive and protective response to overload stress. Nevertheless, persistent pathological myocardial hypertrophy is often accompanied by maladaptive myocardial remodeling. In addition to hypertrophic growth of cardiomyocytes, pressure overload causes a number of other pathological conditions, such as pathological remodeling, activation of cardiac fibroblasts (CF), and rearrangement of the ECM cell matrix. These changes result in fibrosis and impaired cardiac function. Pathological myocardial hypertrophy is an independent risk factor for MI, arrhythmia, chronic heart failure, and sudden death [107].

#### 4.3.2. Long Non-Coding RNAs and Cardiac Hypertrophy

LncRNA has also been determined to play a significant role in the occurrence and progression of cardiac hypertrophy, with more than 1400 lncRNAs identified as deregulated in CFs in hypertrophic mouse hearts [73]. Despite this, there are only a limited number of these lncRNAs for which the function in relation to disease has been identified.

A cluster of lncRNAs partially overlapping the MYH7 gene locus, MHRT (Myosin heavy-chain-associated RNA transcripts), was associated with heart failure [65]. In fact, early studies of lncRNAs related to cardiac hypertrophy found that the lncRNA MHRT protected the heart from cardiac hypertrophy and subsequent heart failure. MHRT is permanently downregulated in a mouse model of TAC surgery. The expression of this lncRNA was accompanied by an isoform switch from MYH6 to MYH7, a hallmark of developing cardiomyopathy [66,67]. Inducible transgenic overexpression of MHRT resulted in reduced cardiac hypertrophy and fibrosis and improved cardiac function compared with mice undergoing TAC surgery without reactivated MHRT [65]. This result was obtained with MHRT overexpression before TAC surgery as well as 2 weeks after the pressure overload initiation. MHRT directly interacted with the chromatin remodeling factor, BRG1 to inhibit its own transcriptional silencing. Conversely, during cardiac stress, BRG1 exceeded MHRT abundance. MHRT binds to the helicase domain of BRG1, which is the major histone acetylation factor, sequesters BRG1 from its genomic DNA targets, and blocks BRG1-driven gene acetylation, which leads to a reduction in the process of cardiac hypertrophy.

CHAST (cardiac hypertrophy-associated transcript) is a pro-hypertrophic lncRNA that promotes hypertrophy [68]. CHAST is highly expressed in the following: (1) Mouse hypertrophic cardiac models [68]; (2) cardiac tissue of patients with aortic stenosis [68]; and (3) cardiomyocytes derived from human embryonic stem cells under the stimulation of hypertrophy. In particular, human CHAST still induces hypertrophic cell growth in murine cardiomyocytes in vitro, suggesting the functional conservation of CHAST in humans and mice. The pro-hypertrophic transcription factor NFAT (nuclear factor of activated T cells) increases CHAST, in which it works by inhibiting the activity of PLEKHM1 (Pleckstrin homology domain-containing protein family M member 1), resulting in autophagy impairment. Adeno-associated virus overexpression of CHAST causes cardiomyocyte hypertrophy both in vitro and in vivo. In contrast, inhibition of CHAST with GapmeR has a cardio-protective effect by preventing hypertrophy and preserving cardiac function in TAC-operated animals. CHAST inhibition before or after hypertrophy would prevent or reduce myocardial hypertrophy, suggesting this lncRNA could potentially become a new therapeutic target.

The heart-enriched lncRNA CHAER (cardiac hypertrophy-associated epigenetic regulator) is also a pre-hypertrophic lncRNA. CHAER is functionally conserved between mice and humans. This lncRNA exemplifies the essential function of lncRNAs in regulating gene expression in response to cardiac stress. Specifically, the mainly nuclear-located CHAER directly interacts with the EZH2 subunit of the PRC2 complex, resulting in reduced histone lysine methylation in promoter regions and thereby enhancing the expression of several pro-hypertrophic genes, such as *ANF*, *MYH7* and *ACTA*. Possibly, the initial interaction between PRC2 and CHAER necessitated the onset of cardiac epigenetic reprogramming but not the progression of hypertrophic remodeling. Conclusions for this hypothesis were obtained when knocking down CHAER was performed 2 days before or 1 day after TAC surgery in rats. Loss of CHAER at an early stage of the disease reduced cardiac hypertrophy and improved cardiac function compared with control animals, whereas knocking down CHAER after 24 h after TAC had no protective effect. However, CHAER knockout in mice resulted in less hypertrophic cardiac growth, reduced fibrosis, and preserved cardiac function after TAC surgery [69], suggesting that CHAER can perform many other functions other than interacting with PRC2. In addition, overexpression of CHAER induced hypertrophic cell growth in cardiomyocytes. Similar to CHAER, MIAT also had a pro-hypertrophic potential in cardiomyocytes. MIAT performed this function by acting as a sponge for two anti-hypertrophic miRNAs, miR-150 and miR-93 [70,71]. The MIAT/miR-150-5p/P300 signaling axis was identified as having an essential role in cardiac hypertrophy in the mouse model with myocardial I/R injury.

Another lncRNA that stimulates hypertrophy is CHRF (Cardiac hypertrophy-related factors). This lncRNA is conserved between humans and mice and acts by sequestering miRNA-489, hence upregulating its downstream target, MYD88, which is a key gene in activating cardiac hypertrophy [72]. The expression of MyD88 is a prerequisite for Ang-II to activate cardiac hypertrophy, and MyD88−/− mice reduce inflammatory responses when treated with Ang-II. CHRF overexpression causes a pathological process of hypertrophy in cardiomyocytes in vitro but induces apoptosis in vivo, suggesting a complex regulatory network of CHRF associated with disease [72,108]. Similar to CHRF, overexpression of the LncRNA MEG3 (Maternally expressed gene 3) also leads to myocardial hypertrophy. MEG3 is the most highly expressed lncRNA and is specific for cardiac fibroblasts. MEG3 was upregulated during the first 4 weeks after TAC, followed by long-term repression. MEG3 helped stabilize or activate p53, which in turn activated MMP-2 (matrix metalloproteinase 2) transcription and modified the ECM composition. Upon MEG3 silencing, pathologic features such as myocardial hypertrophy, fibrosis, and diastolic dysfunction were relieved after 6 weeks in a mouse model of arterial spasm coarctation of the aorta [73].

Several lncRNAs with protective effects on cardiac hypertrophy have also been identified. The lncRNA AHIT (anti-hypertrophy-related transcript) enhances histone methylation, leading to a reduction in stress-induced myocardial hypertrophy in vitro [74]. AHIT binds directly to the promoter of MEF2A, an integral transcription factor in hypertrophy, and recruits SUZ12, a core component of PRC2. The recruited SUZ12 remarkably induces the tri-methylation of the promoter of MEF2A, thereby suppressing MEF2A transcription. Besides AHIT, lncRNA H19, increased in the myocardial hypertrophy mouse model [75], has also been determined to inhibit H3K27 trimethylation, which in turn reverses pathological cardiac hypertrophy [109]. The H19/miR-675/CaMKIId axis is implicated in this process [73]. In addition, mouse, pig, and engineered heart tissue models of cardiac hypertrophy also demonstrate the anti-hypertrophic benefits of H19 [75].

### 4.4. Long Non-Coding RNAs and Heart Failure

#### 4.4.1. Heart Failure

Heart failure is not an independent illness but rather the final stage in the development of many different CVDs. Various pathophysiological stresses induce cardiac remodeling, leading to declined cardiac function, which results in an impaired ability to contract and/or relax. As a result, the heart is not able to perform its full function as a blood pump.

#### 4.4.2. Long Non-Coding RNAs and Heart Failure

Multiple lncRNAs have been implicated in the occurrence and progression of heart failure [110,111]. A study found lncRNAs with changed expression in rats with overload-induced cardiac failure [111]. Interestingly, in this model, lncRNA expression profiles differed with the HF inducers, suggesting that lncRNA expression profiles in HF were aetiologically unique. Data from a study using RNA deep sequencing to compare myocardial tissues from HF patients with left ventricular assist devices and normal controls demonstrated that only lncRNA expression profiles were specific for HF with ischemia origins, while miRNAs and mRNAs could not address this. Another study found 2066 mRNA and 1197 lncRNA increased while 2871 mRNA and 1403 lncRNA decreased in cardiac tissues with ischemic HF [112]. Therein, the expression levels of four pairs of lncRNA-mRNA (MRAK140148-KCND2, MRAK078262-CCRK, MRAK018538-CS, and MRAK053119-Corin) might have a role in the pathogenesis of ischemic HF [112].

Maladaptive cardiac remodeling is a hallmark of HF, and several lncRNAs have been shown to be related to its progression [77,113,114]. As mentioned above, the lncRNA MRHT is a cluster of lncRNAs that partially overlap the Myh7 locus, which contains the human gene encoding β-myosin heavy chain [65,76]. Myosin heavy chain (MHC) is the basic unit of myosin. α-MHC and β-MHC are involved in cardiac contraction [115]. Restoring MRHT expression has cardioprotective capability against pathological hypertrophy and, therefore, against HF [65].

LncRNA BACE1-AS is expressed by all cardiomyocytes. BACE1-AS stabilizes BACE1 mRNA, resulting in its increased expression in cardiomyocytes and Ecs [77]. Both BACE1-AS and BACE1 were raised in HF patients. Likewise, all of these molecules were increased in a mouse model of ischemic HF. BACE1-AS overexpression increased EC apoptosis, and this effect was prevented by silencing BACE1. An increase in BACE1-augmented protein amyloid. Thus, the BACE1-AS/BACE1/β-amyloid pathway contributed to the pathogenesis of HF through the accumulation of β-amyloid which was toxic to ECs and cardiomyocytes.

LncRNA CHRF (Cardiac hypertrophy-related factors) was the first lncRNA associated with HF. In human HF tissues, CHRF was elevated. In the doxorubicin-induced HF model, both CHRF and TGF-β1 were increased in vivo and in vitro. Utilizing valsartan alleviates cardiac dysfunction and injury in HF. However, valsartan no longer had a cardioprotective effect on CHRF overexpression in vivo [78], indicating that valsartan protected against doxorubicin-induced HF in part through a CHRF/TGFβ1 signal pathway [78].

## 5. Potential Utilization of Long Non-Coding RNAs as Biomarkers for Cardiovascular Diseases

Early prediction and diagnosis of CVDs are important to improve patient survival. The biomarkers being used to diagnose CVD are proteins and peptides derived from the heart, for example, troponin T and B-type natriuretic peptides. Not only has it been identified to be involved in the pathogenesis of CVDs, but lncRNAs may also serve as a novel biomarker for non-invasive clinical utilization in the diagnosis and prognosis of CVDs. Compared with other non-coding RNAs, the number of lncRNAs is not abundant, yet they have the right characteristics of a biomarker, including (1) easy access. LncRNA can be detected in extracellular body fluids such as serum, plasma, and urine [97,116,117,118,119,120]; (2) stability. LncRNAs are encapsulated in exosomes, extracellular vesicles [121,122], apoptotic bodies, and proteins that help protect lncRNAs from cleavage by RNAses; and (3) cell type- and disease-specific expression. In other words, lncRNAs can have specific expression patterns in the context of CVDs. These characteristics make them suitable biomarker candidates. In fact, recent clinical trials have been conducted on lncRNAs with the aim of investigating their role as biomarkers in the pathogenesis of some CVDs. Some lncRNAs with diagnostic potential for CVDs are described below.

LncRNA LIPCAR is highly expressed in human plasma samples and has the ability to predict several CVDs, including atherosclerosis, MI, and HF. Among these, LIPCAR has been shown to be an independent predictor of atherosclerosis. Plasma levels of LIPCAR are higher in CAD patients with HF. The level of LIPCAR is correlated with the severity of the clinical outcome [123]. LIPCAR expression was found to be associated with cardiovascular mortality in patients with chronic HF, regardless of pathogenesis [116]. In MI patients, LIPCAR was downregulated early after MI but increased in later stages. In addition, LIPCAR expression levels were related to future maladaptive cardiac remodeling in patients who had experienced an episode of MI. Additionally, the expression of LIPCAR was reduced in patients with ST-segment-elevation MI after coronary angioplasty. This suggested that LIPCAR might serve as a biomarker for ST-segment elevation MI.

CoroMarker (lncRNA OTTHUMT00000387022) from peripheral blood mononuclear cells (PBMCs) was also found to be a good biomarker with high sensitivity and specificity for the diagnosis of atherosclerosis. The expression level of CoroMarker had a positive correlation with genes associated with atherosclerosis. CoroMarker was stable in plasma [121], and removal of CoroMarker reduces the production of proinflammatory cytokines from THP-1 monocytes [124]. However, the exact mechanisms by which CoroMarker regulates monocytes or atherosclerosis have not been determined.

Splicing variants or transcript stability of ANRIL may confer different atherosclerosis susceptibilities in humans. Specifically, analysis of the ANRIL gene region of PBMCs from carriers of the risk haplotype revealed splice variants of ANRIL with distinct expression patterns [125]. The low expression level of the ANRIL splice variant spanning exons 1–2 was strongly correlated with the atherosclerotic phenotype [36]. In addition, ANRIL is an independent risk factor and a good prognostic element for in-stent restenosis. High plasma levels of ANRIL are associated with restenosis within a stent. Besides, ANRIL can also be used as a predictor of ventricular dysfunction after MI [62].

In addition to lncRNAs that have clear potential as biomarkers for CVDs, several other lncRNAs also have potential as biomarkers for CVDs due to an increase or decrease in their association with disease. However, information on the potential of these lncRNAs is still unclear, hence further research is necessary. Specifically, the lncRNA GAS5 controls the interaction of macrophages and ECs during atherosclerotic development, mediated by macrophage-derived exomes following oxLDL stimulation [126]. GAS5 reduces p-mTOR levels in ECs, leading to activation of the proinflammatory response of monocytes/macrophages [127]. LncRNA GAS5 is increased in the plaque of atherosclerosis patients. Nevertheless, the expression level of the lncRNA GAS5 in plasma was significantly lower in atherosclerotic patients. Therefore, more information is needed before this lncRNA can be used in clinical settings. Furthermore, the two lncRNAs NRON (the non-coding repressor of NFAT) and MHRT may be predictors for HF since they have high plasma concentrations in HF patients [128]. MIAT is decreased in PBMCs from MI patients and is related to ST-segment elevation [117]. The specific mitochondrial lncRNAs uc004cov.4 and uc022bqu.1 are enhanced in patients with hypertrophic obstructive cardiomyopathy and can be used as biomarkers for cardiac remodeling in patients with hypertrophic cardiomyopathy [119].

Circulating lncRNAs have great potential for use as molecular markers in the diagnosis and prognosis of CVDs because of their simplicity, stability, and distinct expression in plasma. Thereby, it can help guide clinical decisions that adapt to the individual’s situation. Nevertheless, despite technological advancements, the tools and methods currently used for lncRNAs for clinical purposes lack precision, which explains why they are not widely utilized. Some of the current difficulties include (1) Determining the function and specificity of lncRNAs at various phases of CVD for accurate diagnosis and prognosis is a dilemma. Cardiovascular risk factors, drug use, sex, and age are likely to promote changes in lncRNA expression levels [129,130]; (2) Difficulties in isolating and quantifying. LncRNAs RNA is quite hard to isolate from bodily fluids such as plasma or serum. There are significant differences in the expression levels of lncRNAs between distinct body fluids such as serum, plasma, and urine or even between different compartments of the same cell [119,120]. Technically, there is still an absence of standardization for the isolation, detection, quantification, and normalization of circulating lncRNAs. Criteria regarding liquid samples for circulating lncRNA are not yet available. These are also technical limitations for ncRNAs in general [130,131,132]. (3) The high cost and low throughput of RNA quantification and processing. Currently, lncRNAs cannot replace cardiac troponins in the diagnosis of MI because these clinical tests are relatively cheap and fast. This issue needs to be resolved before lncRNAs can be used in diagnostics.

## 6. The Potential of Utilizing Long Non-Coding RNAs for Therapeutic Applications

Information about the role of lncRNAs in the cardiovascular system and their dysregulation in CVDs is appropriate to consider them as potential molecular targets in therapeutic therapies for CVDs [86,133,134,135]. Abnormal expression of lncRNAs can be resolved by increasing or decreasing their expression levels. Particularly, physiological expression levels of lncRNAs are restored using recombinant plasmids, adenoviruses, or lentiviruses. In contrast, for overexpressed lncRNAs, it is possible to reduce them using shRNA, siRNA [136], aptamers [137], and GapmeR [138] Several studies in animal models have shown positive results. For example, restoring MHRT expression levels resulted in decreased expression of stress-associated genes mediated by BRG1, thus possibly preventing cardiomyopathy [65], or silencing CHAST with GapmeR led to a reduction in TAC-induced cardiac remodeling in a rat model [68]. In particular, there are not any obvious apparent side effects from treatment with GapmeRs. Alternatively, gene-editing tools, such as the CRISPR system, can be used to increase or decrease the expression level of lncRNAs [139,140].

As for pragmatics, modulating the expression levels of cardiac-specific lncRNAs in vivo has shown promising results in terms of ameliorating cardiac dysfunction. But so far, only a few practical examples of therapeutic applications of lncRNAs have been reported. Many issues remain to be overcome before lncRNAs can be applied as a therapeutic approach to CDVs. These issues include (1) lncRNA is still in the process of characterization and annotation. (2) The modulation of a lncRNA in vivo can lead to opposite, even harmful, effects for the primary therapeutic purpose [141], since the same lncRNA can participate in multiple mechanisms of various pathologies [36,142]. (3) The intermediate step between basic research and clinical trials necessarily involves the use of animal models. The lack of gene homology of lncRNAs between different species makes it difficult to transpose results obtained in preclinical studies to humans [143]. Consequently, only identical human lncRNAs can be utilized in final clinical trials. (4) There is still a need to elucidate the secondary and tertiary structures of lncRNAs, as the structure may be more vital for the function of lncRNAs. (5) Issues related to drug delivery to the target lncRNA, such as drug delivery mechanisms, affinity of the therapeutic molecule to the target lncRNA, integrity of its stability in circulation, and pharmacokinetic and pharmacodynamic characterization of the drug. Addressing these issues is critical to the success of lncRNAs as novel therapeutic targets for the treatment of CVD.

## 7. Conclusions

Abnormal expression of lncRNAs is related to CVDs; however, to utilize these molecules in clinical diagnostic, prognostic, and therapeutic applications, there are still many problems to be transcended. Especially, complete information on the properties, functions, and functions of known lncRNAs as well as finding novel lncRNAs in relation to disease will allow more effective applications of this molecule in the future.

## Figures and Tables

**Table 1:** Long non-coding RNAs involved in atherosclerosis, myocardial infarction, cardiac hypertrophy, and heart failure.

No.	LncRNAs	Categories	Target(s)	Mechanism Involved	References
Atherosclerosis					
1	ANRIL	NA	CDKN2A/B miR-199a, miR-125a, miR-186, miR-323		[36,37,38,39,40]
2	TUG1	Intergenic	miR-133a	Depressed expression of FGF1	[41]
3	MEXIS	NA	ABCA1	Increased ABCA1 expression	[42]
Myocardial Infarction					
4	MIAT	Intergenic	miR-24, miR-29, miR-30, miR-133	Increased fibrosis	[43]
5	WISPER	NA	TIAR	Regulated the alternative splicing of PLOD2	[44]
6	H19	Antisense	YB-1 PRC2	Increased expression of COL1A1-mediated H3K27me3, leading to inhibition of TESC activity	[45,46]
7	MIRT	NA	NF-κB		[47,48]
8	APF	NA	miR188	Derepress ATG7 expression	[49]
9	CAIF	NA	P53	Block p53 from activating myocardin transcription	[50]
10	XIST	Intergenic	miR-133a	Derepress SOCS2	[51]
11	CAREL	NA	miR-296	Increased miR-296 target genes TRP53INP1 and ITM2A	[52]
12	CPR	NA	MCM3	Inhibited MCM3, leading to the inhibition of cardiomyocyte proliferation.	[53]
13	CRRL	NA	miR-199a-3p	Inhibited cardiomyocyte regeneration	[54]
14	AZIN2-sv	NA		PI3/Akt signaling pathway	[55]
15	CARL	NA	miR-539	Indirectly increased PHB2 expression, leading to the inhibition of apoptosis in cardiomyocytes.	[56]
16	MDRL	Intergenic	miR-361		[57]
17	FTX	Intergenic	miR-29b	Induced expression of the apoptosis repressor BCL2L2	[58]
18	HOTAIR	Intergenic	miR-1	Releasing BCL2 from miR-1 inhibition, inhibiting program cell death	[59]
19	NRF	NA	miR-873	Releasing RIPK1/RIPK3 results in H_2_O_2_-induced injury attenuation and reduced myocardial ischemic damage	[60]
20	RMRP	Intergenic	miR-206	Enhanced ATG3 and activation of the PI3K/AKT/mTOR pathway	[61]
21	aHIF	Antisense	HIF-1α	Destabilized the HIF-1α mRNA	[62,63]
22	MALAT1	Intergenic	miR-145		[64]
Cardiac Hypertrophy					
23	MHRT	Antisense	Brg1	Prohibit the initiation of the Myh6-to-Myh7 switch; Reduce myocardin acetylation/expression	[65,66,67]
24	CHAST	NA	Plekhm1	Downregulates Plekhm1 to induce the cardiac remodeling processes	[68]
25	CHAER	Intergenic	PCR2	Interact with PCR2 to disinhibit hypertrophic gene expression	[69]
26	MIAT	Intergenic	miR-93	Activate the PI3K/Akt/mTOR pathway via TLR4	[70,71]
27	CHRF	NA	miR-93	Disinhibit the PI3K/Akt pathway	[72]
28	MEG3	Intergenic	miR-361	Derepress HDAC9	[73]
29	AHIT	Antisense	MEF2A	Suppressed MEF2A transcription	[74]
30	H19	Antisense	miR-675	Increased CaMKIId	[75]
Heart Failure					
31	MHRT	Antisense	Brg1	Inhibit the Brg1-Hdac-Parp chromatin repressor complex to prohibit the initiation of the Myh6-to-Myh7 switch; Reduce myocardin acetylation/expression via HDAC5	[65,76]
32	BACE1- AS	Antisense	BACE1	Stabilize BACE1 to increase β-amyloid protein production	[77]
33	CHRF	NA	TGF-β/Smads		[78]

## Data Availability

Not applicable.
